# The Effects of Iterative Freeze–Thaw Cycles on the Structure, Functionality, and Digestibility of *Grifola frondosa* Protein

**DOI:** 10.3390/foods14213608

**Published:** 2025-10-23

**Authors:** Ruiting Li, Zhijie Peng, Sitong Yao, Jialing Huang, Yaxing Wei, Yanfen Cheng, Lijing Xu, Ludan Hou, Junlong Meng, Mingchang Chang, Xueran Geng

**Affiliations:** College of Food Science and Engineering, Shanxi Agricultural University, Taigu, Jinzhong 030801, China; lrtt0625@163.com (R.L.); 15034666080@163.com (Z.P.); yaositong04925@163.com (S.Y.); h2053186174@163.com (J.H.); weiyaxing2023@163.com (Y.W.); cyf2341986@163.com (Y.C.); xulijing383942909@163.com (L.X.); houludan@126.com (L.H.); mengjunlongseth@126.com (J.M.)

**Keywords:** F-T cycle, structural properties, functional properties, in vitro digestion

## Abstract

To improve the processing performance and application potential of *Grifola frondosa* protein (GFP), this study employed multiple freeze–thaw (F-T) cycles to modify GFP and systematically evaluated the changes in its structure, functional properties, and digestive behavior. The results indicated that F-T treatment induced significant oxidation and structural unfolding in GFP, as evidenced by an increase in carbonyl content from 0.75 ± 0.05 nmol/mg to 1.77 ± 0.04 nmol/mg, a decrease in α-helix content from 40.23% to 36.78%, disruption of the microstructure, and degradation of some low-molecular-weight proteins. In terms of functional properties, F-T treatment significantly enhanced the emulsifying performance of GFP, with the best effect observed after 3 F-T cycles. Emulsifying ability increased from 21.83 ± 1.14 m^2^/g to 26.11 ± 1.61 m^2^/g, and emulsifying stability improved from 18.36 ± 1.78% to 25.37 ± 0.84%. This was accompanied by favorable changes in the emulsion’s interfacial properties, including a reduction in average particle size (480.5 ± 30.5 nm) and an increase in absolute ζ-potential (−55.5 ± 0.2 mV). These changes were closely related to the dynamic evolution of free sulfhydryl groups and surface hydrophobicity. In vitro digestion experiments revealed that the protein digestibility and soluble peptide content of GFP peaked after two F-T treatments, reaching 64.88 ± 0.86% and 1.99 ± 0.09118 mg/mL, respectively, and then gradually declined; its antioxidant activity also showed an initial increase followed by a decrease with increasing F-T cycles. In summary, an appropriate number of F-T treatments can effectively enhance the emulsifying properties and digestive characteristics of GFP; this research provides a theoretical basis for the physical modification of GFP and broadens its potential applications in food emulsion products.

## 1. Introduction

The necessity of proteins extends across the entire human lifespan. This is due to their provision of nitrogen and amino acids, which are indispensable substrates for the synthesis of both structural and functional proteins [1]. At present, animal-based proteins continue to be the dominant source of protein in human diets. However, worldwide consumption of these proteins is anticipated to surge, potentially doubling by the year 2050 [2]. In response to growing concerns about environmental impact, health, and global sustainability, the development of novel food resources has accelerated markedly. This shift is exemplified by recent actions from regulatory bodies like China’s National Health Commission (NHC), which has publicized a list of novel foods including *Dendrobium* protocorm, *Pichia kluyveri*, peach gum, tiger nut, and *Leuconostoc mesenteroides* subsp. *Cremoris* [3]. Conventional food production systems are constrained by limited arable land, climate change, and environmental pressures, hindering large-scale protein production enhancement. Therefore, novel alternative protein products will become crucial supplements to human protein sources. Moreover, many alternative proteins offer nutritional profiles comparable to or superior to animal-based proteins [4] while requiring substantially fewer resources and generating lower carbon emissions than conventional livestock farming, representing a pivotal direction for future food systems. A diverse array of alternative protein sources, such as plant-based proteins, insects, edible mushrooms, and algae, are gaining popularity due to their significant potential in addressing future protein needs [5]. From a nutritional standpoint, edible mushrooms represent a superior alternative to animal protein, owing to their distinct advantage of being high in protein while low in fat [6].

Edible mushrooms host a diverse array of essential nutrients, ranging from fundamental vitamins and minerals to valuable bioactive compounds such as polysaccharides, triterpenes, polypeptides, proteins, taurine, and lipids [7]. Edible mushrooms are valued for their appealing aroma and rich nutritional profile—being high in protein, fiber, minerals, and vitamins—coupled with beneficial health attributes, such as low levels of fat and cholesterol, as well as low caloric content. Furthermore, they are associated with a range of health-promoting functions, such as enhancing immunity, lowering blood pressure, and exhibiting broad-spectrum biological activities including antiviral, antibacterial, anti-inflammatory, and anti-tumor effects [8,9]. Fungal proteins from *Grifola frondosa* (maitake) demonstrate particular promise, with both mycelium and fruiting bodies containing 19–40% protein (dry weight basis)—comparable to conventional meat sources and exceeding most plant-based alternatives [10]. This basidiomycete fungus (order: Polyporales) contains 35 g protein/100 g dry matter, doubling the concentration found in Lentinula edodes (shiitake) [11]. *Grifola frondose* is widely recognized for its dual significance, encompassing both substantial nutritional value and notable pharmacological properties [12]. However, the functional properties of proteins are inadequate to meet the actual production and processing needs, such as the poor solubility and emulsification of certain proteins, and they are easily denatured by environmental factors (solution pH, ionic strength, and temperature) during processing [13]. Consequently, the modification of proteins is imperative to enhance their processing characteristics.

Protein modification methods can be broadly categorized into physical, chemical, and enzymatic approaches. As a vital protein modification approach, physical modification employs methods like ultrasonication and thermal treatment to alter protein structures and intermolecular aggregation states, thereby enhancing functional properties. This modification technique offers distinct advantages: low processing costs, high operational safety, short duration, and minimal impact on the nutritional quality of modified products. F-T treatment is a physical modification method involving cyclic ice crystal formation and melting [14]. Freeze–thaw (F-T) cycling is an established technique for modifying starches, including those from potato, wheat, and waxy maize [15,16,17,18]. While the impact of various modification methods on protein structure and functionality has been extensively studied [19,20], the influence of F-T cycles on the structure, functionality, and digestibility of GFP remains largely unexplored.

Existing research on *Grifola frondosa* predominantly focuses on polysaccharide biochemistry [11,21,22], while systematic investigations of its protein components remain limited. In this study, we physically modified *Grifola frondosa* protein (GFP) and investigated the effects of repeated F-T cycles on the alteration of its functional properties and in vitro digestibility. The findings establish a solid theoretical foundation for modifying GFP through controlled F-T cycling, thereby supporting its expanded application in food emulsions and related sectors.

## 2. Materials and Methods

### 2.1. Materials and Reagents

The material used included fruiting bodies of *Grifola frondosa* (containing 19.06% protein and 2.37% fat, purchased from Qingyuan County, Lishui, Zhejiang Province, China) and soybean oil (purchased from Taigu County Agricultural Products Market, Jinzhong, Shanxi Province, China). Sodium hydroxide was purchased from Tianjin Xinbute Chemical Co., Ltd. (Tianjin, China). Tri-glycine and Coomassie Brilliant Blue G-250 was purchased from Beijing Solepol Technology Co., Ltd. (Beijing, China). Trichloroacetic acid (TCA), 2,4-Dinitrophenylhydrazine (DNPH), and 5,5′-dithio bis-(2-nitrobenzoic acid) (DTNB) were purchased from Shanghai Aladdin Biochemical Technology Co. (Shanghai, China). Disodium hydrogen phosphate, sodium dihydrogen phosphate, ethyl acetate, and trichloroacetic acid (TCA) were purchased from Tianjin Kaitong Chemical Reagents Co., Ltd. (Tianjin, China). Every reagent maintained a level of analytical purity.

### 2.2. Preparation of Grifola frondosa Protein

Skimmed *Grifola frondosa* powder was prepared according to the method of Xu et al. with minor modifications [23]. The fruiting bodies of *Grifola frondosa* were first dried at 50 °C for 48 h and ground into a fine powder (WFM-10 Jiangyin Xiangda Machinery Manufacturing Co., Jiangyin, China). Following this, the powder was subjected to a defatting process by mixing it with petroleum ether at a 1:5 (*w*/*v*) ratio for 3 h.

Protein extraction was performed according to Reginio et al. with modifications [24]. The defatted powder was dispersed in deionized water at a 1:30 (*w*/*v*) ratio. The pH of the dispersion was adjusted to 9.0 with 0.5 M NaOH. The mixture was then subjected to ultrasonication (160 W, 40 °C, 3 h) using a KQ55200DE Ultrasonic Cleaner (Kunshan Ultrasonic Instrument Co., Ltd., Kunshan, China), followed by centrifugation at 4800× *g* for 15 min at 25 °C in a Multifuge X1R centrifuge (Thermo Fisher Scientific, Waltham, MA, USA). Subsequently, the supernatant pH was adjusted to 3.5 with 1 M HCl and incubated for 0.5 h at 25 ± 2 °C. The precipitate was centrifuged at 4800× *g* for 15 min, washed three times with deionized water, and adjusted to pH 7.0 with 1 M NaOH. GFP solutions were freeze-dried (Alpha 2-4 LSC; Martin Christ Gefriertrocknungsanlagen GmbH, Osterode am Harz, Germany) and stored in sealed bags at 4 °C until use.

### 2.3. Freeze–Thaw Treatment

**Figure 1:**
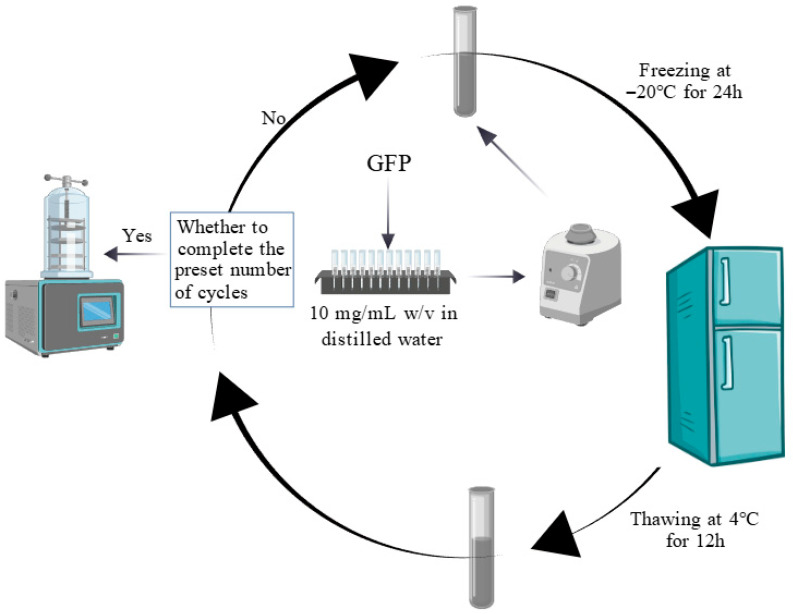
Flowchart of the freeze–thaw cycle for *Grifola frondosa* protein.

The effects of the F-T treatment were measured following the methodology described by Hao et al. with minor modifications [25]. Five GFP samples (10 mg/mL *w*/*v* in distilled water) were dissolved by vortex mixing. Samples were stored at −20 °C until thermal equilibration, followed by F-T cycling. One F-T consisted of freezing at −20 °C for 24 h and thawing at 4 °C for 12 h. After each cycle, samples were collected for lyophilization and analysis, while the remaining solution underwent further cycles. Figure 1 describes this process. This flowchart was created with https://BioGDP.com (accessed on 1 June 2025) [26].

### 2.4. Determination of Free Sulfhydryl Groups

Free sulfhydryl group quantification was performed using a modified Ellman’s protocol [27]. GFP solutions after various F-T cycles were prepared at 3 mg/mL in 0.1 M phosphate buffer (pH 8.0). Then, 30 µL of DTNP (4 mg/mL) was added, and the mixture was vortexed for 5 min. The samples were first incubated in darkness at 20 °C for 30 min and then subjected to centrifugation at 5000 rpm for 15 min. The resulting supernatant was collected, and its absorbance was recorded at 412 nm.

### 2.5. Determination of Protein Carbonyl

The carbonyl content was determined according to the method by Ali et al. with minor modifications [28]. GFP solutions (20 mg/mL) after different F-T cycles were mixed with DNPH solution (10 mM in 2 M HCl) at a 1:20 (*v*/*v*) ratio. After 1 h incubation in darkness, 2 mL of 20% trichloroacetic acid (TCA) was added and vortex-mixed. The mixture was centrifuged at 12,000× *g* for 3 min. The resulting precipitate was subjected to three washes using 2 mL of an ethanol/ethyl acetate mixture (1:1, *v*/*v*). After air-drying, 3 mL of 6 M guanidine hydrochloride was added and vortexed to dissolve the precipitate completely. Carbonyl content (nmol per mg of protein) was determined based on the absorbance measured at 370 nm and an extinction coefficient of 22,000 M^−1^ cm^−1^.

### 2.6. Determination of Surface Hydrophobicity

Surface hydrophobicity was determined according to Vu et al. with modifications [29]. A solution of GFP (5 mg/mL) was prepared by dissolving the samples in 20 mM phosphate buffer (pH 7.0). A 1 mL aliquot of protein solution was mixed with 200 μL of 0.1% (*w*/*v*) bromophenol blue, with phosphate buffer serving as the blank. After vortexing, the mixture was centrifuged at 6500× *g* for 15 min. The supernatant was appropriately diluted and measured at 595 nm using a UV9100A spectrophotometer (LabTech, Beijing, China). Surface hydrophobicity (H_0_) was calculated as follows:

(1) $$\mathrm{Bromophenol}\;\mathrm{blue}\;\mathrm{binding}\;\mathrm{capacity}\left(µg\right)=\frac{200\times \left(A_{0}-A_{1}\right)}{A_{1}}$$

In the equation, A_0_ is the absorbance value of the control group, while A_1_ is the absorbance for the sample group.

### 2.7. Determination of Fourier Transform Infrared Spectroscopy

The secondary structure of the protein samples was characterized by Fourier transform infrared (FTIR) spectroscopy via the KBr tablet method [30]. Protein powder was mixed with potassium bromide (KBr) at a 1:100 (*w*/*w*) ratio. The mixture was ground in an agate mortar and pressed into translucent pellets. A TENSOR 27 spectrometer (Bruker, Karlsruhe, Germany) was employed to record FTIR spectra across the wavenumber range of 400 to 4000 cm^−1^. Deconvolution of the spectra was carried out using OMNIC 9.0 software.

### 2.8. Scanning Electron Microscopy

The microstructure of GFP was observed employing the method adopted from Cui et al. [31]. The protein microstructure was observed using a scanning electron microscope (JSM-6490LV; JEOL Ltd., Tokyo, Japan). Samples were rapidly frozen in liquid nitrogen, freeze-dried under vacuum for 24 h, sputter-coated with gold, and imaged at 3 kV accelerating voltage with 500× magnification.

### 2.9. Sodium Dodecyl Sulfate–Polyacrylamide Gel Electrophoresis

Following Zang et al. with modifications [32], GFP samples were dissolved in deionized water at 2 mg/mL. Protein solutions were mixed with Laemmli sample buffer at a 5:1 (*v*/*v*) ratio. The mixture was loaded onto a polyacrylamide gel (12% separating gel, 5% stacking gel), and electrophoresis was performed. The gel was stained with Coomassie Brilliant Blue R-250 and destained in 40% methanol/10% acetic acid overnight. Images were captured using a gel documentation system.

### 2.10. Determination of Solubility

The solubility of MP was determined according to the method described by Zhou et al. [33]. GFP samples (0.1 g) after 0–5 F-T cycles were dissolved in deionized water to a final concentration of 2 mg/mL. After adjusting pH to 7.0 with 0.1 M NaOH or HCl, suspensions were vortexed for 5 min and centrifuged at 6500× *g* for 10 min. Supernatant protein content was measured at 595 nm using the Bradford assay with Coomassie Brilliant Blue G-250. Protein solubility was then defined by the following equation:

(2) $$\mathrm{Solubility}\left(\%\right)=\frac{\mathrm{Protein}\;\mathrm{Content}\;\mathrm{in}\;\mathrm{the}\;\mathrm{Supernatant}}{\mathrm{Protein}\;\mathrm{Content}\;\mathrm{of}\;\mathrm{the}\;\mathrm{Sample}}\times 100$$

### 2.11. Determination of Water-Holding Capacity and Oil-Holding Capacity

The water-holding and oil-holding capacities were measured as described by Tang et al. [34]. F-T-treated GFP samples (0.1 g) were weighed into preweighed centrifuge tubes (recorded as m_1_). Deionized water (2 mL) was added and vortexed at 25 °C for 5 min. After centrifugation at 6500× *g* for 10 min, supernatants were decanted. Tubes containing precipitates were weighed (m_2_). Water-holding capacity (WHC, %) was calculated as follows:

(3) $$\mathrm{Water}\text{-}\mathrm{holding}\;\mathrm{capacity}\left(\%\right)=\frac{m_{2}-m_{1}}{m}\times 100$$

Oil-holding capacity (OHC) was determined similarly using soybean oil (density 0.92 g/mL). F-T-treated GFP samples (0.1 g) were weighed into preweighed centrifuge tubes (recorded as m_1_), mixed with 5 mL soybean oil, and vortexed at 25 °C for 5 min. After centrifugation at 6500× *g* for 10 min, free oil was removed by inverting tubes for 5 min. Tubes containing pellets with bound oil were weighed (m_2_). Oil-holding capacity (OHC, g oil/g protein) was calculated as follows:

(4) $$\mathrm{Oil}\text{-}\mathrm{holding}\;\mathrm{capacity}\left(\%\right)=\frac{m_{2}-m_{1}}{m}\times 100$$

In the equation, m is the mass of the protein sample (g); m_1_ is the mass of the centrifuge tube with the protein sample (g); and m_2_ is the mass of the centrifuge tube with the precipitate (g).

### 2.12. Determination of Foaming Properties

The foaming ability (FA) and foaming stability (FS) of GFP were measured according to the method described by Hu et al. [35]. F-T-treated GFP was dissolved in deionized water at 10 mg/mL. A 20 mL aliquot of protein solution was transferred to a 100 mL centrifuge tube. The solution was homogenized at 10,000× *g* for 3 min using a high-speed homogenizer. Foam volume (V_0_) was immediately measured. After 10 min quiescence at 25 ± 1 °C, the foam volume was remeasured (V_10_). Foaming capacity (%) and foaming stability (%) were calculated as follows:

(5) $$\mathrm{Foaming}\;\mathrm{ability}\left(\%\right)=\frac{V_{0}}{V}\times 100$$

(6) $$\mathrm{Foaming}\;\mathrm{stability}=\frac{V_{10}}{V_{0}}\times 100$$

In the equation, V is the volume of the original protein solution (mL); V_0_ is the foam volume immediately after homogenization (mL); and V_10_ is the foam volume after 10 min (mL).

### 2.13. Determination of Emulsifying Properties

Emulsifying ability index (EAI) and emulsion stability index (ESI) were evaluated for GFP with reference to a published method [36]. GFP samples were dissolved in deionized water at 10 mg/mL. Protein solution (20 mL) was mixed with 5 mL soybean oil in 100 mL centrifuge tubes. Emulsions were formed by high-speed homogenization at 10,000× *g* for 3 min. Immediately after homogenization, 20 μL aliquots were collected from the emulsion middle layer and mixed with 5 mL of 0.1% SDS (*w*/*v*) by vortexing. Absorbance at 500 nm (A_0_) was measured using 0.1% SDS as a blank. After 10 min quiescence at 25 °C, duplicate 20 μL aliquots were similarly processed and measured (A_10_). Emulsifying ability index (m^2^/g) and emulsion stability index (min) were calculated as follows:

(7) $$\mathrm{Emulsifying}\;\mathrm{ability}\left(\frac{m_{2}}{g}\right)=\frac{2\times 2.303\times N\times A_{0}}{C\times \varphi \times L\times 10^{4}}$$

(8) $$\mathrm{Emulsifying}\;\mathrm{stability}\left(\%\right)=\frac{A_{0}\times 10}{A_{0}-A_{10}}\times 100$$

In the aforementioned equation, N is the dilution factor, C is the sample concentration (g/mL), φ is the proportion of the oil phase in the emulsion, and L is the light path of the cuvette (mm).

### 2.14. Particle Size and Zeta Potential

GFP samples were dissolved in deionized water at 10 mg/mL. Protein solution (20 mL) was mixed with 5 mL soybean oil in 100 mL centrifuge tubes. Emulsions were formed by high-speed homogenization at 10,000× *g* for 3 min, filtered through 0.45 μm membranes, and diluted 100-fold with distilled water. The particle size and ζ-potential of the GFP composite nanoparticles were assessed according to the method of Zhang et al. [37]. Particle size and ζ-potential measurements were performed at 25 °C on a Zetasizer Nano ZS90 (Malvern Panalytical, Malvern, UK), after allowing the samples to equilibrate for 2 min. The refractive indices for the solute and dispersant (water, pH 7.0) were set at 1.334 and 1.330, respectively. Each reported value represents the mean of three independent measurements.

### 2.15. In Vitro Digestion

The freeze-dried samples underwent in vitro simulated digestion as described by Zhu et al. [38].

Simulated gastric fluid preparation included the following. A total of 900 mg NaCl was dissolved in 100 mL PBS (0.2 mol/L, pH 7.2). The pH was adjusted to 2.0 with HCl, followed by the addition of 300 mg pepsin. The solution was stirred until complete dissolution.

Simulated intestinal fluid preparation included the following. A total of 1 g porcine bile salts and 300 mg trypsin were dissolved in 100 mL PBS (0.2 mol/L, pH 7.2). The pH was adjusted to 8.0 with NaOH, and the solution was equilibrated.

The digestion protocol was as follows. A freeze-dried sample (200 mg) was added to 10 mL simulated gastric fluid and shaken at 37 °C for 2 h. After centrifugation at 8000× *g* for 2 min, the supernatant (gastric digest) was collected. The precipitate was resuspended in 10 mL simulated intestinal fluid, shaken at 37 °C for 2 h, and then centrifuged at 8000× *g* for 2 min. The supernatant (intestinal digest) was collected, and enzymes were inactivated by boiling for 10 min.

### 2.16. Measurement of Protein Digestibility

Protein digestibility was determined following the method of Zhou et al. [39]. Briefly, a 5 mL digest sample was combined with an equal volume of 10% (*v*/*v*) trichloroacetic acid (TCA). Prior to centrifugation at 12,000× *g* for 5 min, the mixture was kept at 4 °C overnight to facilitate protein precipitation. The supernatant was collected, and its protein content was quantified. Digestibility (%) was calculated as follows:

(9) $$\mathrm{Digestibility}\%=\frac{\mathrm{Protein}\;\mathrm{Content}\;\mathrm{in}\;\mathrm{the}\;\mathrm{Supernatant}}{\mathrm{Protein}\;\mathrm{Content}\;\mathrm{in}\;\mathrm{the}\;\mathrm{Sample}}\times 100$$

### 2.17. Determination of Peptide Content

The peptide content was quantified following the method of Yan et al. [40], with slight adaptations. A 2.5 mL digestate was mixed with an equal volume (2.5 mL) of 10% (*v*/*v*) trichloroacetic acid (TCA). After 10 min of incubation at 25 °C, the mixture was centrifuged at 6500× *g* for 15 min. The supernatant was collected and diluted to 50 mL with 5% TCA. Subsequently, 6 mL of this diluted solution was transferred to a test tube, mixed with 4 mL of ninhydrin reagent by vortexing, and incubated at 25 °C for 10 min. The solution was centrifuged at 5000× *g* for 10 min, and the supernatant absorbance was measured at 540 nm. The peptide content must be calculated using the standard curve.

**Figure 2:**
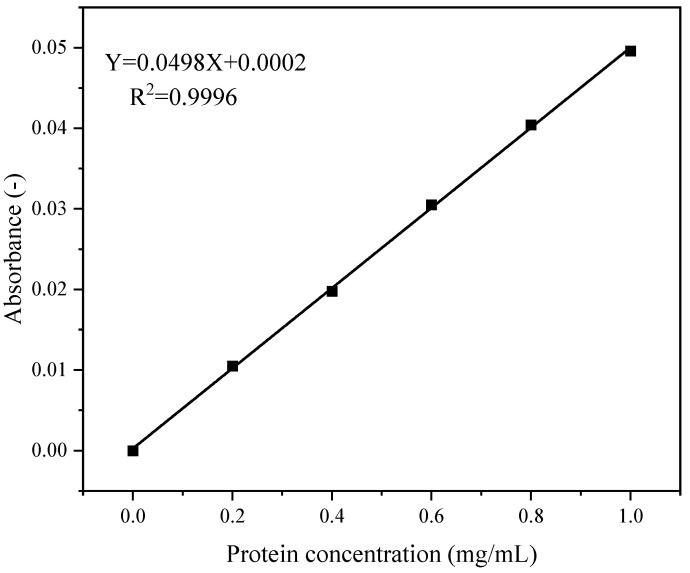
Standard curve for peptide content.

Figure 2 shows the standard curve for peptide content quantification. The regression equation was y = 0.0498x + 0.0002, with a coefficient of determination (R^2^) of 0.9996.

### 2.18. DPPH Radical Scavenging Capacity

The DPPH radical scavenging capacity was assessed following the method of Liu, Y. et al. [41], with slight modifications. The GFP digestates from different freeze–thaw cycles were mixed with a 0.1 mM DPPH ethanol solution in a 1:1 (*v*/*v*) ratio. After reacting in darkness at 25 °C for 30 min, the absorbance was measured at 517 nm against a solvent blank (distilled water replaced the sample). The DPPH radical scavenging capacity (%) was calculated as follows:

$$\mathrm{DPPH}\;\mathrm{Radical}\;\mathrm{Scavenging}\;\mathrm{rate}\%=1-\frac{A_{1}-A_{2}}{A_{0}}\times 100\;$$

where A_1_ corresponds to the absorbance of the test group, A_2_ refers to the control group absorbance, and A_0_ signifies the blank group measurement.

### 2.19. Hydroxyl Radical Scavenging Capacity

The hydroxyl radical (•OH) scavenging capacity was assessed following the method of Liu et al. [41], with slight modifications. The GFP digestate samples, following different freeze–thaw cycles, were mixed with FeSO_4_, salicylic acid, and 30% H_2_O_2_ in a 1:1:1 ratio. The mixture was then incubated at 37 °C for 30 min. The absorbance was measured at 510 nm against a blank (distilled water replaced the sample). The hydroxyl radical scavenging capacity (%) was calculated as follows:

$$•\mathrm{OH}\;\mathrm{Scavenging}\;\mathrm{rate}\%=1-\frac{A_{1}-A_{2}}{A_{0}}\times 100$$

where A_1_ corresponds to the absorbance of the test group, A_2_ refers to the control group absorbance, and A_0_ signifies the blank group measurement.

### 2.20. Determination of Reducing Capacity

The reducing capacity was assessed following the method of Tsuda et al. [42], with slight modifications. Digestate samples (1 mL) from different freeze–thaw cycles were mixed with an equal volume (1 mL) of 1% (*w*/*v*) potassium ferricyanide. The mixture was incubated at 50 °C for 20 min. After incubation, 1 mL of 10% (*w*/*v*) trichloroacetic acid (TCA) was added. The solution was then rapidly cooled to 25 °C and centrifuged at 12,000× *g* for 2 min. Subsequently, a 1 mL aliquot of the supernatant was mixed with 1 mL of distilled water and 0.2 mL of 0.1% (*w*/*v*) FeCl_3_. After reacting for 10 min at 25 °C, the absorbance was measured at 700 nm against a distilled water blank. Reducing power was calculated as follows:

$$\mathrm{Reducing}\;\mathrm{capacity}=A_{1}-A_{2}-A_{0}$$

In the equation, A_1_ corresponds to the test group absorbance, A_2_ refers to the control group value, and A_0_ denotes the blank group reading.

### 2.21. Determination of Metal Chelating Capacity

A modified version of the assay described by Pérez-Burillo et al. [43] was used to determine the metal chelating ability. A total of 1 mL of digestate from various freeze–thaw cycles was mixed with 3.7 mL of distilled water, 0.1 mL of FeCl_2_ solution, and 0.2 mL of ferrozine solution. The resulting mixture was incubated at 25 °C for 10 min.

$$\mathrm{Scavenging}\;\mathrm{rate}\%=1-\frac{A_{1}-A_{2}}{A_{0}}\times 100$$

In the equation, A_1_ corresponds to the test group absorbance, A_2_ refers to the control group value, and A_0_ denotes the blank group reading.

### 2.22. Statistical Analysis

All experiments were conducted with three independent replicates. Data are expressed as mean ± standard deviation and were analyzed using IBM SPSS Statistics 27.0 (IBM Corp., Armonk, NY, USA). Significant differences among groups were evaluated by one-way analysis of variance (ANOVA) coupled with Duncan’s post hoc test, with a significance level of *p* < 0.05. Differences between groups are indicated by different superscript letters. Graphical representations of the data were generated using Origin 2018. (OriginLab Corporation, Northampton, MA, USA).

## 3. Results and Discussion

### 3.1. Free Sulfhydryl Analysis

**Figure 3:**
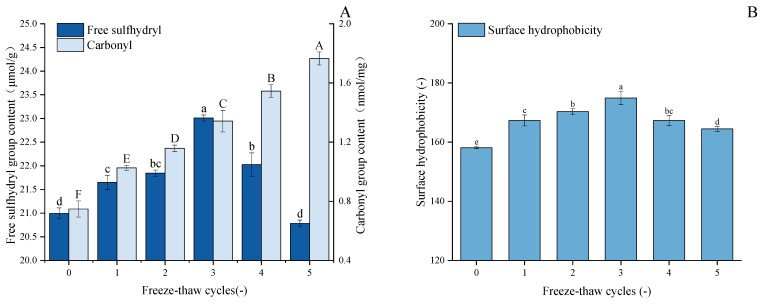
Effects of freeze–thaw cycles on the structural properties of *Grifola frondosa* protein. (**A**) Changes in free sulfhydryl content (lowercase letters) and carbonyl content (uppercase letters). (**B**) Changes in surface hydrophobicity (lowercase letters). Different letters indicate statistically significant differences (*p* < 0.05) among different F-T cycles.

As demonstrated in Figure 3A, with an increase in F-T cycles, the free sulfhydryl content of GFP undergoes significant changes, exhibiting a trend of initial gradual increase followed by rapid decline. After three freeze–thaw cycles, the free sulfhydryl content in GFP increased by 9.6% to 23.01 μmol/g protein. During the initial stage of freeze–thaw cycles, disulfide bonds were cleaved, leading to an increase in free sulfhydryl levels through bond dissociation. However, when the number of freeze–thaw cycles increased to a certain extent, external environmental stimuli caused oxidation of free sulfhydryl groups, promoting the formation of new disulfide bonds and consequently leading to a decrease in sulfhydryl content [44]. Hence, the free sulfhydryl content of GFP decreased during the fourth and fifth freeze–thaw cycles. This observation is supported by previous work on peanut protein isolate, which reported analogous trends in free sulfhydryl group changes upon F-T treatment [45]. Furthermore, during F-T processes, the redistribution of water molecules and ice recrystallization phenomena may also affect the availability of protein sulfhydryl groups [46].

### 3.2. Carbonyl Analysis

The carbonyl content in GFP increased progressively during five freeze–thaw cycles (Figure 3A), rising from an initial value of 0.755 nmol/mg protein to 1.77 nmol/mg protein after the fifth cycle, which corresponds to a 134% increase. The potential underlying cause of this phenomenon is the repeated formation and melting of ice crystals, accompanied by the increased volume of ice crystals, which, in turn, result in mechanical damage to protein structures. During freezing and thawing, the formation of ice crystals and changes in the microenvironment within the protein structure may promote the production of reactive oxygen species, which further trigger oxidative reactions by targeting specific amino acid side chains such as arginine, lysine, and proline, leading to the formation of carbonyl residues [47]. Similar patterns of carbonyl group changes in myofibrillar protein after F-T cycles were reported by Sun et al. [48]. An additional contribution to the elevated carbonyl levels may stem from the oxidative cleavage of peptide bonds [49].

### 3.3. Surface Hydrophobicity Analysis

Protein surface hydrophobicity (H_0_) is a quantitative measure of the hydrophobic groups exposed by the protein surface when interacting with a polar aqueous environment. As shown in Figure 3B, F-T cycling significantly enhanced the surface hydrophobicity of GFP compared to the native state (*p* < 0.05). The H_0_ value peaked at 174.95 µg after three cycles (from a baseline of 158.13 µg) and subsequently decreased to 164.47 µg after five cycles, without dropping below the initial level. As established, freezing and thawing induce the partial unfolding of protein molecules, thereby exposing buried hydrophobic regions and increasing surface hydrophobicity [50]. The initial rise in hydrophobicity observed after repeated F-T cycles can be attributed to this molecular rearrangement and the exposure of hydrophobic side chains, as reported by Zhang et al. [51]. However, with excessive F-T cycles, the unfolded proteins become more susceptible to oxidation upon contact with water molecules and ice crystals, leading to a certain degree of aggregation [28]. Consequently, the surface hydrophobicity (H_0_) of GFP increased to a peak before subsequently declining. This parameter is critical as it reflects the protein’s capacity to anchor at the oil–water interface via hydrophobic interactions, which is fundamental to its emulsifying properties [52].

### 3.4. Fourier Transform Infrared Spectroscopy Analyses

**Figure 4:**
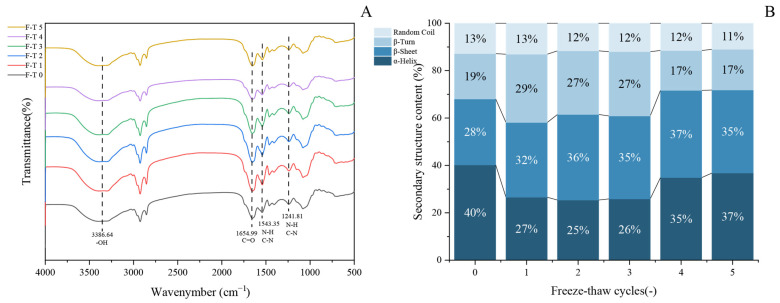
Evolution of secondary structure in *Grifola frondosa* protein during freeze–thaw cycling. (**A**) Fourier transform infrared spectroscopy showing structural changes. (**B**) Quantitative analysis of secondary structure content derived from amide I band deconvolution.

The secondary structure represents the most fundamental configuration of proteins and serves as a key indicator of their structural changes. Fourier transform infrared (FTIR) spectroscopy is widely used to characterize protein molecular conformations based on their absorption at specific wavelengths. This technique has been proven particularly effective in identifying characteristic functional groups and molecular structures [53]. As shown in Figure 4A, the FTIR spectrum of GFP displays characteristic absorption bands at 3386.64 cm^−1^, 1654.99 cm^−1^, and 1543.35 cm^−1^, which are attributed to O–H stretching vibration, C = O stretching vibration, and the combination of C–N stretching and N–H bending vibrations, respectively [54]. The overall spectral profiles of untreated and freeze–thaw-treated GFP are largely consistent.

To gain deeper insight into changes in the secondary structure, the amide I band was processed by Gaussian fitting and deconvolution using OMNIC software (Thermo Fisher Scientific, Waltham, MA, USA, version 8.2), in accordance with established secondary structure assignments for specific spectral regions. The content of each secondary structure was calculated based on the relative area of its corresponding peak. In the amide I band, the regions 1646–1664 cm^−1^, 1615–1637 cm^−1^, 1682–1700 cm^−1^, 1664–1681 cm^−1^, and 1637–1645 cm^−1^ are characteristic of α-helix, β-sheet, β-turn, and random coil structures, respectively. Quantitative analysis of the amide I band (Figure 4B) revealed dynamic changes in the secondary structure of GFP after F-T cycles [55]. During the initial cycles (1–3), the α-helix content decreased significantly, accompanied by an increase in β-sheet structures, indicating a partial loosening of the native protein conformation. The reduction in α-helix content led to the exposure of hydrophobic regions, consistent with the observed rise in surface hydrophobicity (Figure 3B). In subsequent cycles (4–5), the α-helix content showed a partial recovery, though it remained lower than that of the untreated protein, while the β-sheet content also increased relative to the native state. This structural shift can be attributed to the propensity of freeze–thaw treatment to promote the conversion of α-helices to β-sheets [56] and potentially to the initiation of new β-sheet structures through interactions among exposed hydrophobic groups [57].

### 3.5. Sodium Dodecyl Sulfate–Polyacrylamide Gel Electrophoresis Analysis

**Figure 5:**
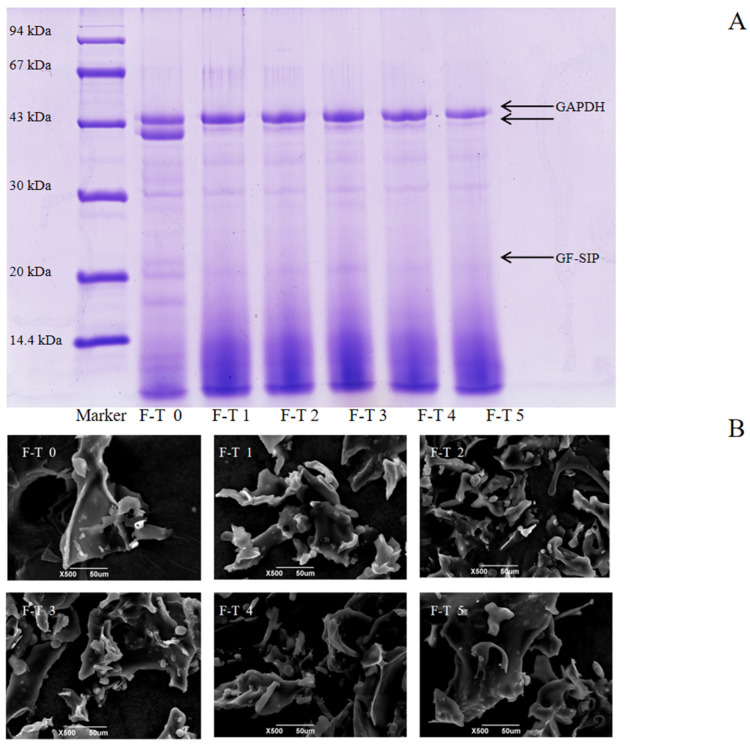
Molecular weight distribution and microstructure of *Grifola frondosa* protein (GFP) under freeze–thaw (F-T) cycles. (**A**) Sodium dodecyl sulfate–polyacrylamide gel electrophoresis after different F-T cycles. (**B**) Scanning electron microscopy images of GFP after different F-T cycles. Scale bar: 50 μm.

Proteins with different molecular weights can be separated by SDS gel electrophoresis [58]. Analysis of the sodium dodecyl sulfate–polyacrylamide gel electrophoresis (SDS-PAGE) results in Figure 5A revealed that the untreated protein exhibited two distinct bands with molecular weights around 43 kDa, along with several fainter bands near 30 kDa and 20 kDa. In contrast, proteins subjected to 1–5 freeze–thaw cycles showed only one clear, prominent band at 43 kDa, as the second 43 kDa band gradually faded with increasing cycles. Moreover, the bands near 30 kDa and 20 kDa also diminished, with individual bands gradually fading or disappearing. A noticeable smearing effect also appeared at the bottom of the gel (low-molecular-weight region). These phenomena collectively reveal the dual damaging effects of freeze–thaw cycles on proteins. On one hand, the proteins undergo nonspecific degradation, generating numerous fragments of varying molecular weights, which leads to the smearing in the low-molecular-weight region. On the other hand, repeated F–T cycles caused protein aggregation [59], forming insoluble macromolecular complexes that cannot enter the separating gel. As demonstrated by Jiang et al. [60], the redistribution of water molecules and the recrystallization of ice during the F-T process can oxidize proteins and even produce denaturation or irreparable mechanical damage. Consequently, the protein structure is damaged and degraded.

### 3.6. Scanning Electron Microscopy Analysis

Scanning electron microscopy (SEM) was employed to visualize the microstructural evolution of GFP under F-T stress, as shown in Figure 5B. The untreated GFP exhibited a flaky, wavy morphology. This structure reflects the homogeneous nature of the native protein prior to F-T treatment. Following 1–3 F-T cycles, this cohesive structure disintegrated into a loose, fragmented state with diverse morphologies (flakes, globules, rods). This disruption is a direct consequence of freeze concentration. The formation of ice crystals excludes proteins into a confined space, dramatically increasing their local concentration and promoting molecular collisions. Concurrently, the mechanical shear from ice crystals disrupts the native protein structure, exposing hydrophobic regions previously buried in the interior. This leads to aberrant protein–protein interactions and the formation of disordered, insoluble aggregates, manifesting as the observed fragments and rough surfaces. After 4–5 F-T cycles, the microstructure reconsolidated into larger, coarser aggregates with reduced fine debris. This indicates a secondary process where the initially formed small aggregates further coalesced and reorganized via strong hydrophobic interactions.

### 3.7. Protein Solubility Analysis

**Figure 6:**
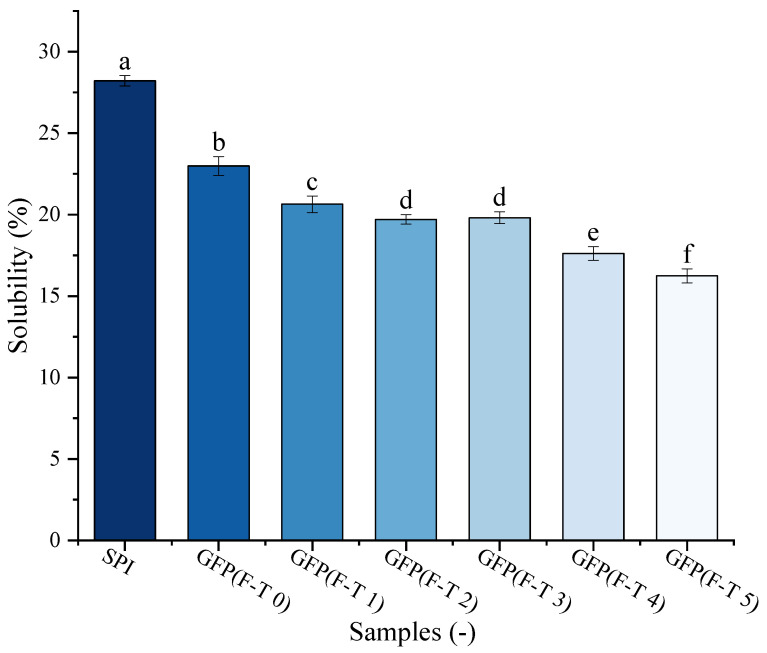
Solubility of *Grifola frondosa* protein (GFP) after 0–5 freeze–thaw (F-T) cycles compared with soy protein isolate (SPI). Different lowercase letters indicate statistically significant differences (*p* < 0.05) in solubility among all samples (including both GFP at different cycles and SPI).

Protein solubility is one of the crucial GFP functional features. The solubility of GFP under F-T treatment was evaluated against a commercial soy protein isolate (SPI) as a benchmark (Figure 6). The solubility of untreated GFP (22.99%) was lower than that of SPI (28.22%). Following F-T treatment, GFP exhibited a pronounced and progressive decline in solubility, significantly dropping to 16.24% after five cycles. This may be attributed to oxidative processes, such as aggregation, cross-linking, and cleavage occurring between protein molecules as F-T cycles increase, which destabilize the protein matrix and consequently reduce solubility [61]. As reported by Cao et al. [62], freezing imposes multiple stresses—including ice crystal formation and the cryo-concentration of solutes and buffers—that can induce protein denaturation. Such denatured proteins subsequently undergo aggregation or even precipitation, ultimately leading to a decline in solubility [63].

### 3.8. Water-Holding Capacity Analysis

**Figure 7:**
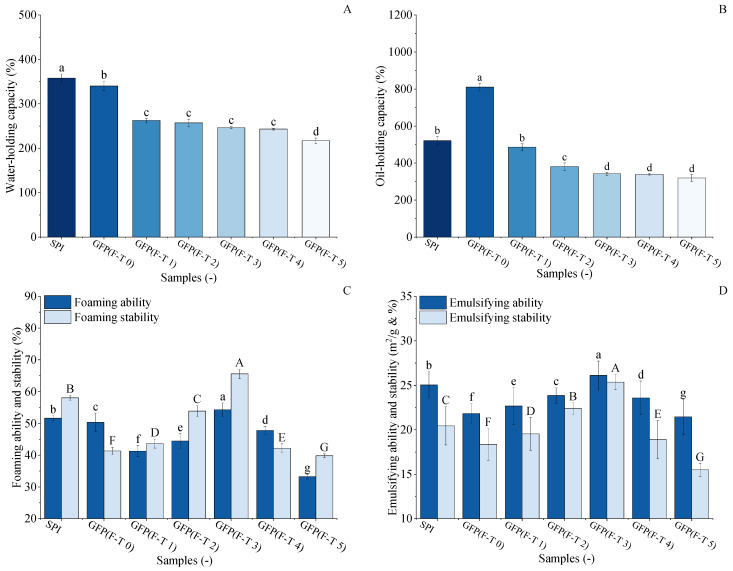
Comparative analysis of functional properties between *Grifola frondosa* protein (GFP) under freeze–thaw (F-T) and soy protein isolate (SPI). (**A**) Water-holding capacity. (**B**) Oil-holding capacity. (**C**) Foaming properties. Foaming capacity (lowercase letters) and foaming stability (uppercase letters). (**D**) Emulsifying properties. Emulsifying capacity (lowercase letters) and emulsion stability (uppercase letters). Different letters indicate statistically significant differences (*p* < 0.05).

The WHC of GFP under F-T treatment was evaluated and compared with SPI, as shown in Figure 7A. The initial WHC of untreated GFP (340.15%) was slightly lower than that of SPI (357.83%). After 1–4 F-T cycles, the WHC of GFP decreased to 243.13%, followed by a marked decline to 216.77% after the fifth cycle. This final value represents a 36.2% reduction from the initial WHC of GFP and a 39.4% reduction relative to the SPI benchmark. The decrease in WHC may be due to the growth and recrystallization of ice crystals [64], which leads to the loosening of spatial structural connections and the disruption of integrity, resulting in a decrease in water-holding capacity [65].

### 3.9. Oil-Holding Capacity Analysis

The oil-holding capacity (OHC) of GFP under freeze–thaw (F-T) treatment was evaluated against soy protein isolate (SPI), as shown in Figure 7B. Untreated GFP exhibited a remarkably high OHC of 810.4%, significantly surpassing that of SPI (520.63%). However, this advantage diminished rapidly upon F-T treatment. After just one cycle, GFP’s OHC dropped to 485.81%, falling below the SPI benchmark, and it continued to decline to 319.05% after five cycles, representing a 60.6% reduction from its initial value. This substantial loss indicates GFP’s structural vulnerability to freezing and thawing processes. During freezing and thawing, the external environment of proteins is in a process of constant and drastic changes, during which both the spatial structure and morphology of proteins change, making proteins denatured and less capable of interacting with lipid molecules, affecting their OHC [66].

### 3.10. Foaming and Emulsifying Analysis

The foaming properties of GFP were evaluated and compared with those of soy protein isolate SPI to assess its functional potential and response to F-T treatment (Figure 7C). While the untreated GFP exhibited lower initial FA (50.34%) and FS (43.33%) compared to SPI (FA, 60.33%; FS, 41.35%), its performance was markedly enhanced by moderate F-T cycling. The FA of GFP initially increased with F-T cycles, peaking at 54.32% after the third cycle, while its FS simultaneously reached a maximum of 65.50%. Crucially, after three F-T cycles, both the FC and FS of GFP surpassed the corresponding values of the untreated SPI. This reversal demonstrates that controlled F-T cycling constitutes a viable physical modification method for GFP. The improvement is likely due to the F-T-induced partial unfolding and structural loosening of GFP, which enhances its flexibility and adsorption capacity at the air–water interface, thereby facilitating foam formation and stability [67]. The subsequent decline in later cycles indicates an optimal modification window before excessive damage occurs.

The emulsifying properties of GFP were evaluated and compared with those of SPI to assess its practical potential, as shown in Figure 7D. The untreated GFP had an emulsifying ability of 21.83 m^2^/g and a stability of 18.36%, which were lower than those of SPI (26.05 m^2^/g and 20.44%). However, during F-T treatment, GFP’s emulsifying ability and stability exhibited a distinct initial rise followed by a decline, both peaking after the third cycle at 26.11 m^2^/g and 25.37%, respectively. Crucially, these optimized values not only represented a significant improvement for GFP but also surpassed the baseline performance of the untreated SPI. This demonstrates that controlled F-T stress can effectively enhance GFP’s emulsifying functionality to a level competitive with a commercial soy protein. As suggested by Kato et al. [53], this improvement is likely due to the F-T-induced exposure of hydrophobic groups, which enhances the protein’s capacity to adsorb at oil–water interfaces and form reinforced interfacial layers.

### 3.11. Particle Size and Zeta Potential Analysis

Table 1 presents the average droplet size and zeta potential—a key indicator of emulsification performance—of the GFP-stabilized emulsion. In the dynamic light scattering (DLS) measurements, all particle sizes are reported as the volume-weighted mean. The mean droplet diameters of the emulsions subjected to zero to five freeze–thaw (F-T) cycles were 1261 ± 20.1 nm, 832.1 ± 32.8 nm, 695.5 ± 19.4 nm, 480.5 ± 30.5 nm, 718.3 ± 57.5 nm, and 1089 ± 35.6 nm, respectively. Notably, all F-T-treated samples exhibited significantly smaller particle sizes compared to the untreated control (0 cycle). The initial decrease in droplet size reached a minimum after the third cycle, a trend consistent with the emulsification capacity. This improvement can be attributed to the partial unfolding of GFP induced by F-T treatment, which increased surface hydrophobicity and enhanced protein adsorption at the the oil–water interface—a factor positively correlated with emulsification performance [45]. Beyond the third cycle, however, the particle size increased markedly, suggesting a reversal of the initial improvement. This shift likely results from oxidative damage leading to excessive protein aggregation. Our data support this mechanism; the decline in free sulfhydryl groups and the rise in carbonyl content after the fourth and fifth cycles indicate substantial protein oxidation. Pearson correlation analysis was employed to quantitatively link these oxidative modifications to the observed particle size increase. The analysis revealed a highly significant negative correlation between particle size and free sulfhydryl content (r = −0.932, *p* < 0.01). This strong correlation underscores that the loss of sulfhydryl groups, which can lead to disulfide bond formation and altered protein interactions, is a primary driver of the aggregation responsible for larger droplet sizes.

In contrast, the zeta potential of the emulsions exhibited a trend inverse to that of particle size. The recorded values from zero to five cycles were −39.8 ± 1.2 mV, −46.1 ± 3.2 mV, −47.7 ± 2.9 mV, −55.5 ± 0.2 mV, −49.6 ± 3.0 mV, and −47.9 ± 2.1 mV, respectively. Importantly, all F-T-treated emulsions showed significantly higher absolute zeta potential values than the untreated control, indicating enhanced electrostatic repulsion between oil droplets. The maximum zeta potential observed at the third cycle reflects an optimal level of protein structural rearrangement, positioning more charged groups at the interface. The subsequent decline in zeta potential likely stems from the formation of large aggregates that shield charged residues, leading to a less uniform and effective interfacial layer. According to McClements and Li [68], specific molecular rearrangements at the interface can augment surface charge. The strengthened electrostatic repulsion in treated emulsions helps overcome attractive forces between droplets, thereby improving emulsion stability and mitigating flocculation and coalescence [69,70].

### 3.12. Digestibility Analysis

**Figure 8:**
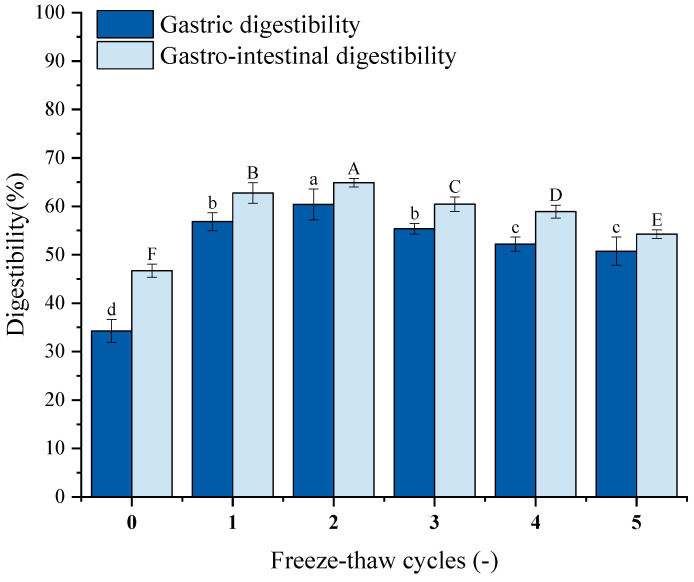
Effects of freeze–thaw (F-T) cycles on *Grifola frondosa* protein (GFP) digestibility. Gastric digestibility (lowercase letters) and gastrointestinal digestibility (uppercase letters). Different letters indicate statistically significant differences (*p* < 0.05).

Protein digestibility is an essential factor that affects amino acids accessibility [59], which may reflect the nutritional quality of food. Figure 8 illustrates the impact of repeated F-T cycles on GFP digestibility. Both gastric and gastrointestinal digestibility after five cycles were significantly higher than untreated controls. Gastrointestinal digestibility exceeded gastric digestibility. Gastric digestibility increased from 34.28% to 56.83%, 60.40%, 55.36%, 52.17%, and 50.74%; gastrointestinal digestibility increased from 46.71% to 62.75%, 64.88%, 62.45%, 58.90%, and 54.26%. Studies reveal that ice crystal formation/melting during F-T disrupts hydration layers around polar groups, inducing unfolding of higher-order structures [71]. This may expose the digestive enzyme’s cleavage site and enhance the enzyme’s ability to bind to it, resulting in increased digestibility. Excessive cycling promotes aggregation of unfolded proteins via hydrophobic interactions [72].

### 3.13. Polypeptide Content Analysis

**Figure 9:**
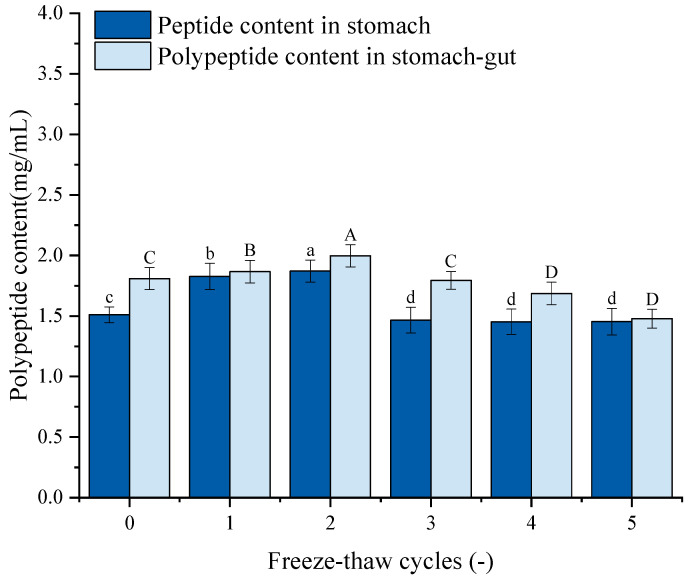
Effects of freeze–thaw (F-T) cycles on peptide content from *Grifola frondosa* protein (GFP) digestates. Peptide content after gastric digestion (lowercase letters) and gastrointestinal digestion (uppercase letters). Different letters indicate statistically significant differences (*p* < 0.05).

Figure 9 depicts the peptide content of GFP following simulated gastrointestinal digestion. The peptide content exhibited a trend of initial increase followed by a decrease, mirroring the pattern of protein digestibility. Specifically, after 1–2 F-T cycles, the peptide content increased significantly from the untreated control (1.51 mg/mL and 1.81 mg/mL) to 1.87 mg/mL and 1.99 mg/mL, respectively. This initial increase suggests that moderate F-T stress partially unfolds the protein, enhancing its susceptibility to proteolytic enzymes. However, after 3–5 F-T cycles, the peptide content declined, dropping to 1.45 mg/mL and 1.48 mg/mL after the fifth cycle. This decrease is likely attributed to the excessive protein aggregation and oxidative damage induced by intense F-T treatment. Severe F-T cycles promote the formation of large, densely packed aggregates with potentially cross-linked structures. These aggregates are physically less accessible to digestive proteases.

### 3.14. DPPH Radical Scavenging Capacity Analysis

**Figure 10:**
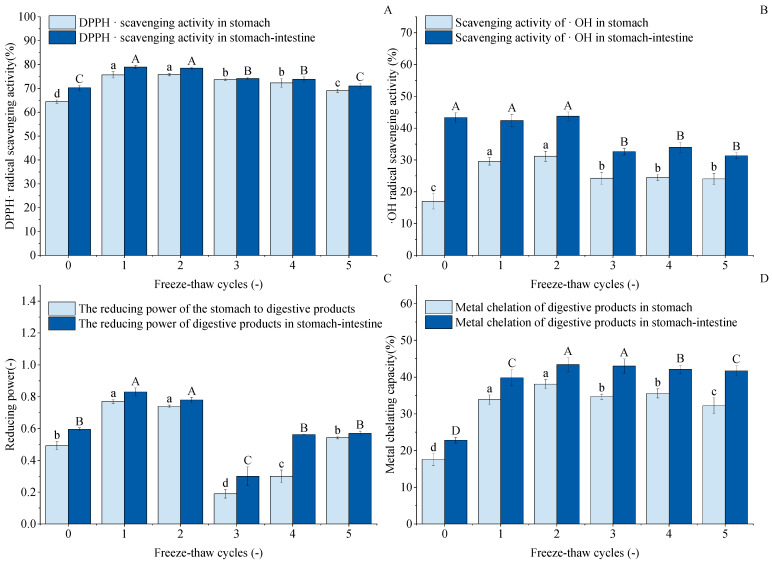
Effects of freeze–thaw (F-T) cycles on antioxidant capacity of *Grifola frondosa* protein (GFP) digestates. (**A**) DPPH· radical scavenging activity. (**B**) ·OH radical scavenging activity. (**C**) Reducing power. (**D**) Metal chelating capacity. Different lowercase letters indicate statistically significant differences (*p* < 0.05) in gastric digestates, while different uppercase letters indicate statistically significant differences (*p* < 0.05) in gastrointestinal digestates.

Figure 10A shows the DPPH· scavenging capacity of digested GFP after F-T cycles. The figure indicates that after 1–5 cycles, both gastric and gastrointestinal digests exhibited higher DPPH· scavenging capacity than untreated controls. Gastric scavenging capacity increased from an initial 64.47% to 75.72%, 75.80%, 73.66%, 72.34%, and 69.01%, respectively. Cycle 1 and 2 results were comparable with no significant difference. After gastrointestinal digestion, the trend of DPPH· scavenging capacity was not obvious; the highest scavenging rate was 78.96% at the first time, but there was no difference between the two compared to the second time. The results of 3~5 F-T cycles were reduced by 74.11%, 73.87%, and 71.02%, respectively, but increased by 3.87%, 3.63%, and 0.78% compared to the untreated group. This indicates that F-T treatment enhanced the DPPH· scavenging capacity of GFP digests.

### 3.15. Hydroxyl Radical Scavenging Capability Analysis

As shown in Figure 10B, the hydroxyl radical scavenging capacity (-OH scavenging capacity) of the gastric digests from freeze–thaw-treated GFP was significantly enhanced compared to the untreated control (16.99%). This suggests that moderate freeze–thaw treatment induces partial protein unfolding, which may expose encapsulated antioxidant amino acid residues and facilitate their hydrolysis and release by pepsin, thereby generating more small-molecule peptides with antioxidant activity. However, after 3–5 freeze–thaw cycles, the -OH scavenging capacity of both the gastric and intestinal digests decreased significantly and then plateaued, with no significant differences observed among these groups. This decline can be attributed to the excessive protein aggregation and oxidation induced by repeated freeze–thaw treatments, as supported by our earlier structural characterization. The dense aggregates formed are likely resistant to enzymatic hydrolysis. As a consequence, the antioxidant peptides were hard to release into the digestion product [73,74]. Consequently, although the intestinal digests generally exhibited higher capacity than the gastric phase due to more complete hydrolysis, excessive processing ultimately diminished the potential of GFP to yield antioxidant peptides.

### 3.16. Reducing Capacity Analysis

As shown in Figure 10C, the reducing capacity of the GFP digest showed a unique nonlinear trend in response to F-T cycles, which differed from other antioxidant indices. The reduced capacity increased after one F-T cycle (peaking at 0.77 and 0.83). This may be because moderate F-T stress induces partial protein unfolding, exposing more enzyme cleavage sites or originally encapsulated reducing amino acid residues, resulting in the release of more small reducing peptides during digestion. However, the sharp decrease observed at the third F-T cycle marks a critical point, suggesting that while slight oxidation enhances the reducing capacity, excessive oxidation leads to a significant decrease [75]. The subsequent slight recovery in cycles 4–5 may result from a state of severe aggregation and oxidation, in which certain otherwise deeply embedded and strongly reducing groups are exposed due to the complete disruption of the protein structure.

### 3.17. Metal Chelating Capacity Analysis

The metal chelating capacity of proteins is a key contributor to their overall antioxidant capacity. As shown in Figure 10D, this capacity in the GFP digest was significantly enhanced by F-T treatment, reaching a peak after the second cycle. This enhancement can be attributed to the F-T-induced structural changes of native GFP, where the exposure of internal hydrophobic groups and regions played a key role [76]. The subsequent decrease in chelating capacity after excessive cycling (3–5) may be due to the onset of severe aggregation and oxidation, which can bury these functional residues in insoluble complexes or chemically modify them, thus reducing their chelating potential.

## 4. Conclusions

Our findings demonstrate that repeat F-T cycles significantly alter the structure, functionality, and digestibility of GFP. The process is characterized by an initial phase of partial unfolding (after 1–3 cycles), which enhances surface hydrophobicity and improves foaming and emulsifying properties to a level surpassing a commercial soy protein isolate. Beyond this point, excessive F-T treatment leads to protein aggregation, which disrupts microstructure, reduces solubility, and diminishes functional properties. The digestibility and antioxidant capacity of GFP during simulated digestion were also found to be governed by this structural transition. This study elucidates the modification mechanism of GFP during the F-T process, thereby establishing a theoretical foundation for its future development and potential utilization in food systems.

## Figures and Tables

**Table 1:** Effect of freeze–thaw cycling on the mean particle size, PDI, and ζ-potential of *Grifola frondosa* protein-stabilized emulsions.

	Mean Particle Size (nm)	Polydispersity Index (PDI)	Zeta Potential (Mv)
F-T 0	1261 ± 20.1 ^e^	0.273 ± 0.074	−39.8 ± 1.2 ^D^
F-T 1	832.1 ± 32.8 ^c^	0.294 ± 0.039	−46.1 ± 3.2 ^C^
F-T 2	695.5 ± 19.4 ^b^	0.399 ± 0.081	−47.7 ± 2.9 ^C^
F-T 3	480.5 ± 30.5 ^a^	0.369 ± 0.077	−55.5 ± 0.2 ^A^
F-T 4	718.3 ± 57.5 ^b^	0.636 ± 0.033	−49.6 ± 3.0 ^B^
F-T 5	1089 ± 35.6 ^d^	0.402 ± 0.026	−47.9 ± 2.1 ^C^

Notes: Different lowercase letters within the same column indicate statistically significant differences (*p* < 0.05) in particle size among different F-T cycles, while different uppercase letters within the same column indicate statistically significant differences (*p* < 0.05) in ζ-potential among different F-T cycles.

## Data Availability

The original contributions presented in the study are included in the article; further inquiries can be directed to the corresponding authors.

## Abbreviations

The following abbreviations are used in this manuscript:

GFP	*Grifola frondosa* protein
Freeze–thaw	F-T
WHC	Water-holding capacity
OHC	Oil-holding capacity
FC	Foaming capacity
FS	Foaming stability
EAI	Emulsifying ability index
ESI	Emulsion stability index
FTIR	Fourier transform infrared spectroscopy
SEM	Scanning electron microscopy
SPI	Soy protein isolate
ANOVA	One-way analysis of variance
GMM	Gaussian multi-peak fitting

## References

[1] Montebello R., Derossi A., Caporizzi R., Taherzadeh M.J., Rousta N., Severini C. (2025). Pooling scientific information on the nutritional, sensory, and technological properties of mycoprotein to support its role in creating a more sustainable food system. Food Biosci..

[2] Pojić M., Mišan A., Tiwari B. (2018). Eco-innovative technologies for extraction of proteins for human consumption from renewable protein sources of plant origin. Trends Food Sci. Technol..

[3] Guo Q., Zhang M., Mujumdar A.S., Yu D. (2024). Drying technologies of novel food resources for future foods: Progress, challenges and application prospects. Food Biosci..

[4] Parodi A., Leip A., Boer I.J.M., Slegers P.M., Ziegler F., Temme E., Herrero M., Tuomisto H., Valin H., Middelaar C. (2018). The potential of future foods for sustainable and healthy diets. Nat. Sustain..

[5] Pastrana-Pastrana Á.J., Rodríguez-Herrera R., Solanilla-Duque J.F., Flores-Gallegos A.C. (2025). Plant proteins, insects, edible mushrooms and algae: More sustainable alternatives to conventional animal protein. J. Future Foods.

[6] Mishra A., Shankar S. (2025). Edible mushrooms for improved human health, food security and environmental sustainability: A critical review. Sci. Total Environ..

[7] Barros L., Venturini B.A., Baptista P., Estevinho L.M., Ferreira I.C. (2008). Chemical composition and biological properties of portuguese wild mushrooms: A comprehensive study. J. Agric. Food Chem..

[8] Ionescu M., Dincă M.-N., Ferdeș M., Zăbavă B.-Ș., Paraschiv G., Moiceanu G. (2025). Proteins from Edible Mushrooms: Nutritional Role and Contribution to Well-Being. Foods.

[9] Lindequist U., Niedermeyer T.H., Jülich W.D. (2005). The pharmacological potential of mushrooms. Evid. Based Complement. Altern. Med..

[10] Olson B., Marks D.L., Grossberg A.J. (2020). Diverging metabolic programmes and behaviours during states of starvation, protein malnutrition, and cachexia. J. Cachexia Sarcopenia Muscle.

[11] Song T., Zhang T., Cai Q., Ding Y.-Y., Gu Q., Gu Z. (2024). The Grifola frondosa-derived ACE inhibitory peptide attenuated the exosomes-mediated phenotype transformation of vascular smooth muscle cells. J. Funct. Foods.

[12] Jang S., Park S., Park G., Ku B., Kim M., Kang J., Oh S., Han H., Kim S., Lee H. (2025). Grifola frondosa (Maitake) extract as natural antioxidant on emulsion-type pork sausages. Food Chem. X.

[13] Li Q., Wang Z., Dai C., Wang Y., Chen W., Ju X., Yuan J., He R. (2019). Physical stability and microstructure of rapeseed protein isolate/gum Arabic stabilized emulsions at alkaline pH. Food Hydrocoll..

[14] Wang L., Xie B., Xiong G., Wu W., Wang J., Qiao Y., Liao L. (2013). The effect of freeze–thaw cycles on microstructure and physicochemical properties of four starch gels. Food Hydrocoll..

[15] Liu M., Ma H., Liang Y., Sun L., Li J., Dang W., Li L., Zheng X., Lv Q., Zhang X. (2022). Effect of multiple freezing/thawing cycles on the physicochemical properties and structural characteristics of starch from wheat flours with different gluten strength. Int. J. Biol. Macromol..

[16] Martínez P., Betalleluz-Pallardel I., Cuba A., Peña F., Cervantes-Uc J.M., Uribe-Calderón J.A., Velezmoro C. (2022). Effects of natural freeze-thaw treatment on structural, functional, and rheological characteristics of starches isolated from three bitter potato cultivars from the Andean region. Food Hydrocoll..

[17] Wang M., Bai X., Jiang Y., Lang S., Yu L. (2019). Preparation and characterization of low oil absorption starch via freeze-thawing. Carbohydr. Polym..

[18] Yu S., Zhang Y., Li H., Wang Y., Gong C., Liu X., Zheng X., Kopparapu N.K. (2015). Effect of freeze-thawing treatment on the microstructure and thermal properties of non-waxy corn starch granule. Starch Starke.

[19] Dong X., Zhao M., Shi J., Yang B., Li J., Luo D., Jiang G., Jiang Y. (2011). Effects of combined high-pressure homogenization and enzymatic treatment on extraction yield, hydrolysis and function properties of peanut proteins. Innov. Food Sci. Emerg. Technol..

[20] Jamdar S.N., Rajalakshmi V., Pednekar M.D., Juan F., Yardi V., Sharma A. (2010). Influence of degree of hydrolysis on functional properties, antioxidant activity and ACE inhibitory activity of peanut protein hydrolysate. Food Chem..

[21] Arsiccio A., Giorsello P., Marenco L., Pisano R. (2020). Considerations on Protein Stability During Freezing and Its Impact on the Freeze-Drying Cycle: A Design Space Approach. J. Pharm. Sci..

[22] Minatovicz B., Sun L., Foran C., Chaudhuri B., Tang C., Shameem M. (2020). Freeze-concentration of solutes during bulk freezing and its impact on protein stability. J. Drug Deliv. Sci. Technol..

[23] Xu L., Kumar A., Lamb K. (2004). A laboratory study for developing an aqueous process to make skimmed soymilk. J. Am. Oil Chem. Soc..

[24] Reginio F., Wei Q., Ketnawa S., Ogawa Y. (2020). Bio-properties of Saba banana (Musa ‘saba’, ABB Group): Influence of maturity and changes during simulated in vitro gastrointestinal digestion. Sci. Rep..

[25] Hao Q., Lei Y., Li R., Ma L., Zheng H., Deng X., Zhang J. (2024). The effect of freeze–thaw cycles on the physicochemical stability and nutritional composition of camel milk. LWT.

[26] Jiang S., Li H., Zhang L., Mu W., Zhang Y., Chen T., Wu J., Tang H., Zheng S., Liu Y. (2025). Generic Diagramming Platform (GDP): A Comprehensive Database of High-quality Biomedical Graphics. Nucleic Acids Res..

[27] Wang P., Chen H., Mohanad B., Xu L., Ning Y., Xu J., Wu F., Yang N., Jin Z., Xu X. (2014). Effect of frozen storage on physico-chemistry of wheat gluten proteins: Studies on gluten-, glutenin- and gliadin-rich fractions. Food Hydrocoll..

[28] Ali S., Zhang W., Rajput N., Khan M.A., Li C.-B., Zhou G.-H. (2015). Effect of multiple freeze–thaw cycles on the quality of chicken breast meat. Food Chem..

[29] Vu T.-H., Bean S., Hsieh C.-F., Shi Y.-C. (2017). Changes in protein and starch digestibility in sorghum flour during heat-moisture treatments. J. Sci. Food Agric..

[30] Wang C., Li T., Ma L., Li T., Yu H., Hou J., Jiang Z. (2020). Consequences of superfine grinding treatment on structure, physicochemical and rheological properties of transglutaminase-crosslinked whey protein isolate. Food Chem..

[31] Cui B., Zeng X., Liang H., Li J., Zhou B., Wu D., Du X., Li B. (2024). Construction of a soybean protein isolate/polysaccharide-based whole muscle meat analog: Physical properties and freeze-thawing stability study. Int. J. Biol. Macromol..

[32] Zang X., Yue C., Wang Y., Shao M., Yu G. (2019). Effect of limited enzymatic hydrolysis on the structure and emulsifying properties of rice bran protein. J. Cereal Sci..

[33] Zhou Y., Xu Y., Yu D., Wang B. (2024). Effects of freeze-thaw cycles and heat treatment on the odor-binding properties of myofibrillar protein to key fishy compounds. Food Biosci..

[34] Tang W., Ye L., Han T., He J., Liu J. (2024). Effect of Chitosan with Different Molecular Weights on the Freeze-Thaw Stability of Gluten Protein: Protein Structures, Functional Characteristics, and Cryo-Protective Mechanism. Food Hydrocoll..

[35] Hu F., Zou P.-R., Zhang F., Thakur K., Khan M.R., Busquets R., Zhang J.-G., Wei Z.-J. (2022). Wheat gluten proteins phosphorylated with sodium tripolyphosphate: Changes in structure to improve functional properties for expanding applications. Curr. Res. Food Sci..

[36] Wang L., Wu M., Liu H.-M. (2017). Emulsifying and physicochemical properties of soy hull hemicelluloses-soy protein isolate conjugates. Carbohydr. Polym..

[37] Zhang Y., Xie Y., Qiu M., He H., Liao D., Zhao H., Hu G., Geng F. (2025). Insight into the difference in nutritional yolk granules of different poultry eggs from the perspective of quantitative lipidomics combined with nutrient analysis. Food Sci. Hum. Wellness.

[38] Zhu Y., Lei W., Qiu L., Liu S., Guo R., Huang Y., Liu L., Lv M., Sun B., Qu M. (2025). Effect of freezing-thawing treatment on the quality and structure of soymilk gels induced by different coagulants. Food Hydrocoll..

[39] Zhou S.-D., Lin Y.-F., Xu X., Meng L., Dong M.-S. (2020). Effect of non-covalent and covalent complexation of (−)-epigallocatechin gallate with soybean protein isolate on protein structure and in vitro digestion characteristics. Food Chem..

[40] Yan Z.-F., Yuan S., Qin Q., Wu J. (2022). Enhancement of rice protein hydrolysate quality using a novel dual enzyme system. LWT.

[41] Liu Y., Li X., Qin H., Huang M., Xi B., Mao J., Zhang S. (2024). Comparing the antioxidation and bioavailability of polysaccharides from extruded and unextruded Baijiu vinasses via in vitro digestion and fecal fermentation. Int. J. Biol. Macromol..

[42] Tsuda T., Watanabe M., Ohshima K., Norinobu S., Choi S.W., Kawakishi S., Osawa T. (1994). Antioxidative activity of the anthocyanin pigments cyanidin 3-O-beta-D-glucoside and cyanidin. J. Agric. Food Chem..

[43] Pérez-Burillo S., Mehta T., Pastoriza S., Kramer D.L., Paliy O., Rufián-Henares J.Á. (2019). Potential probiotic salami with dietary fiber modulates antioxidant capacity, short chain fatty acid production and gut microbiota community structure. LWT.

[44] Zhao L., Li L., Liu G.-Q., Liu X.-X., Li B. (2012). Effect of frozen storage on molecular weight, size distribution and conformation of gluten by SAXS and SEC-MALLS. Molecules.

[45] Feng H., Jin H., Gao Y., Yan S., Zhang Y., Zhao Q., Xu J. (2020). Effects of freeze-thaw cycles on the structure and emulsifying properties of peanut protein isolates. Food Chem..

[46] Wang P., Jin Z., Xu X. (2015). Physicochemical alterations of wheat gluten proteins upon dough formation and frozen storage—A review from gluten, glutenin and gliadin perspectives. Trends Food Sci. Technol..

[47] Bao Y., Ertbjerg P., Estévez M., Yuan L., Gao R. (2021). Freezing of meat and aquatic food: Underlying mechanisms and implications on protein oxidation. Compr. Rev. Food Sci. Food Saf..

[48] Sun Y., Li X., Liu H., Chai Y., Bao Y., Li F. (2025). The impact of Pleurotus eryngii on myofibrillar protein: Physicochemical properties and structural alterations in quick-frozen pork patties during freeze-thaw cycles. Food Chem. X.

[49] He Y., Huang H., Li L., Yang X., Hao S., Chen S., Deng J. (2018). The effects of modified atmosphere packaging and enzyme inhibitors on protein oxidation of tilapia muscle during iced storage. LWT Food Sci. Technol..

[50] Zhang Y., Kim Y.H.B., Puolanne E., Ertbjerg P. (2022). Role of freezing-induced myofibrillar protein denaturation in the generation of thaw loss: A review. Meat Sci..

[51] Zhang R., Li S., Ai M., Chen S. (2025). Pickering emulsions stabilized by ultrasound-assisted phosphorylated cantaloupe seed protein isolate −chitosan: Preparation, characterization and stability. Ultrason. Sonochem..

[52] Zhang Q.-T., Tu Z.-C., Xiao H., Wang H., Huang X.-Q., Liu G.-X., Liu C.-M., Shi Y., Fan L.-L., Lin D.-R. (2014). Influence of ultrasonic treatment on the structure and emulsifying properties of peanut protein isolate. Food Bioprod. Process..

[53] Kato A., Nakai S. (1980). Hydrophobicity determined by a fluorescence probe method and its correlation with surface properties of proteins. Biochim. Biophys. Acta (BBA) Protein Struct..

[54] Dai L., Sun C., Wei Y., Zhan X., Mao L. (2018). Formation and characterization of zein-propylene glycol alginate-surfactant ternary complexes: Effect of surfactant type. Food Chem..

[55] Wan W., Feng J., Wang H., Du X., Wang B., Yu G., Xia X. (2023). Influence of repeated freeze-thaw treatments on the oxidation and degradation of muscle proteins from mirror carp (*Cyprinus carpio* L.), based on myofibrillar protein structural changes. Int. J. Biol. Macromol..

[56] Khan A.N., Khan R.H. (2022). Protein misfolding and related human diseases: A comprehensive review of toxicity, proteins involved, and current therapeutic strategies. Int. J. Biol. Macromol..

[57] Williams A.D., Portelius E., Kheterpal I., Guo J.-T., Cook K.D., Xu Y., Wetzel R. (2004). Mapping Aβ Amyloid Fibril Secondary Structure Using Scanning Proline Mutagenesis. J. Mol. Biol..

[58] Ramírez-Guerra H., García-Sifuentes C., Pacheco-Aguilar R., Ramírez-Suárez J. (2012). The influence of ante-mortem hypoxia on the physicochemical stability of myofibrillar proteins in the muscle tissue of white shrimp (*Litopenaeus vannamei*) exposed to multiple freeze–thaw cycles. Eur. Food Res. Technol..

[59] Bai X., Shi S., Kong B., Chen Q., Liu Q., Li Z., Wu K., Xia X. (2023). Analysis of the influencing mechanism of the freeze–thawing cycles on in vitro chicken meat digestion based on protein structural changes. Food Chem..

[60] Jiang Q., Nakazawa N., Hu Y., Osako K., Okazaki E. (2019). Microstructural modification and its effect on the quality attributes of frozen-thawed bigeye tuna (*Thunnus obesus*) meat during salting. LWT.

[61] Rahman M.H., Hossain M.M., Rahman S.M., Amin M.R., Oh D.H. (2015). Evaluation of Physicochemical Deterioration and Lipid Oxidation of Beef Muscle Affected by Freeze-thaw Cycles. Korean J. Food Sci. Anim. Resour..

[62] Cao E., Chen Y., Cui Z., Foster P. (2003). Effect of freezing and thawing rates on denaturation of protein in aqueous solutions. Biotechnol. Bioeng..

[63] Tang C.-H., Wang X.-Y., Yang X.-Q., Li L. (2009). Formation of soluble aggregates from insoluble commercial soy protein isolate by means of ultrasonic treatment and their gelling properties. J. Food Eng..

[64] Du X., Chang P., Tian J., Kong B., Sun F., Xia X. (2020). Effect of ice structuring protein on the quality, thermal stability and oxidation of mirror carp (*Cyprinus carpio* L.) induced by freeze-thaw cycles. LWT.

[65] Suresh Kumar K., Ganesan K., Selvaraj K., Rao P.V.S. (2014). Studies on the functional properties of protein concentrate of *Kappaphycus alvarezii* (Doty) Doty—An edible seaweed. Food Chem..

[66] Jiang J., Zhu B., Liu Y., Xiong Y.L. (2014). Correction to Interfacial Structural Role of pH-Shifting Processed Pea Protein in the Oxidative Stability of Oil/Water Emulsions. J. Agric. Food Chem..

[67] Jambrak A.R., Lelas V., Mason T.J., Krešić G., Badanjak M. (2009). Physical properties of ultrasound treated soy proteins. J. Food Eng..

[68] McClements D.J., Li Y. (2010). Structured emulsion-based delivery systems: Controlling the digestion and release of lipophilic food components. Adv. Colloid. Interface Sci..

[69] Cho H.T., Salvia-Trujillo L., Kim J., Park Y., Xiao H., McClements D.J. (2014). Droplet size and composition of nutraceutical nanoemulsions influences bioavailability of long chain fatty acids and Coenzyme Q10. Food Chem..

[70] Qiu C., Zhao M., Decker E.A., McClements D.J. (2015). Influence of protein type on oxidation and digestibility of fish oil-in-water emulsions: Gliadin, caseinate, and whey protein. Food Chem..

[71] Yamashita Y., Zhang N., Nozaki Y. (2003). Effect of chitin hydrolysate on the denaturation of lizard fish myofibrillar protein and the state of water during frozen storage. Food Hydrocoll..

[72] Santé-Lhoutellier V., Engel E., Aubry L., Gatellier P. (2008). Effect of animal (lamb) diet and meat storage on myofibrillar protein oxidation and in vitro digestibility. Meat Sci..

[73] Ballatore M.B., Bettiol M.D.R., Braber N.L.V., Aminahuel C.A., Rossi Y.E., Petroselli G., Erra-Balsells R., Cavaglieri L.R., Montenegro M.A. (2020). Antioxidant and cytoprotective effect of peptides produced by hydrolysis of whey protein concentrate with trypsin. Food Chem..

[74] Dorman H.J.D., Peltoketo A., Hiltunen R., Tikkanen M.J. (2003). Characterisation of the antioxidant properties of de-odourised aqueous extracts from selected *Lamiaceae herbs*. Food Chem..

[75] Zhang H., Yu X., You J., Xiong S., Liu Y. (2024). Effects of hydroxyl radicals oxidation on digestion properties of silver carp myofibrillar protein gel. LWT.

[76] Zhao L., Cheng X., Song X., Ouyang D., Wang J., Wu Q., Jia J. (2023). Ultrasonic assisted extraction of mulberry leaf protein: Kinetic model, structural and functional properties, in vitro digestion. Process Biochem..

